# Clinical and immunologic profile of children and adolescents submitted to cow milk oral immunotheray (OIT)

**DOI:** 10.1186/1939-4551-8-S1-A185

**Published:** 2015-04-08

**Authors:** Conrado Martins, Alfredo Alves Neto, Fabio Kuschnir, José Luiz De Magalhães Rios, Marilucia Alves Da Venda, João Bosco Magalhães Rios

**Affiliations:** 1 Policlinica Geral Do Rio De Janeiro,Brazil; 2 Faculdade De Medicina De Petropolis - Fase, Brazil; 3 Rio De Janeiro State University, Brazil

## Background

The Paradigm of cow’s milk allergy (CMA) management has shifted in the last years, with the introduction of the OIT protocols to CMA. The purpose of this research is to describe clinical and immunological characteristics of patients who underwent CMA OIT, to delineate a profile of these patients.

## Methods

Case series involving 15 children over 4 years and adolescents who still had anaphylaxis to Cow's Milk. We assessed their socio-demographic characteristics, the clinical profile of manifestations and immunological findings, before beginning the OIT.

## Results

The mean age was 8.73 years (min: 4, Max: 19), and 9 were females. The symptoms started before 1 year old in 93% of the sample and in 50% before 6 months. Urticaria was the most frequent manifestation in the 1st contact (60%), some progressing to anaphylaxis: the initial event in 40% of cases. Half of the patients responded to minimal amounts of LV, mainly (60%) in raw state. Symptoms appeared in less than 1 hour in 96%. Until the beginning of treatment 73% have had 1-5 episodes of anaphylaxis. About 75% of patients have associated respiratory allergy and 33% had some anaphylactic event in the last 12 months. The specific IgE levels were elevated in 93% of the sample: For full LV the mean level was 50.2 KU/L and the median 41 KU/L (Min: 1.4 KU/l and Max: >100 KU/L); for casein, the mean was 40 KU/L, and the median 28.4 KU/L (0.8 - >100). For α-lacto albumin and β-lacto globulin, these values were respectively: 23.1 KU/L, 12.1 KU/L (0.4 - 82) and 9.94 KU/L, 4:46 KU/L (0.7 - 31.4).

## Conclusions

Severe forms of CMA initiates early (1st year old) and can persist through adolescence. The most frequent initial manifestation is urticaria, which appears less than 1 hour after ingestion of small quantities. The levels of specific IgE to CM proteins are usually very high, especially casein.

